# Superior Primary Stability of a Knotless Double‐Row Construct Compared to Mason‐Allen Repair for Anatomical Refixation of Gluteal Tendons—Biomechanical Human Cadaver Study

**DOI:** 10.1111/os.70153

**Published:** 2025-08-13

**Authors:** Vanessa Twardy, Daniela Warnecke, Peter M. Prodinger, Norbert Harrasser, Christian Scheele, Rüdiger von Eisenhart‐Rothe, Martina Roth, Ingo J. Banke

**Affiliations:** ^1^ Clinic of Orthopedics and Sports Orthopedics, Klinikum Rechts der Isar, Technical University of Munich Munich Germany; ^2^ Orthopedic Research Department, Arthrex GmbH Munich Germany; ^3^ Medical Care Center Holzkirchen GmbH Holzkirchen Germany

**Keywords:** anatomical refixation, gluteal tendon repair, gluteus medius, gluteus minimus, greater trochanteric pain syndrome (GTPS), HipBridge, SpeedBridge

## Abstract

**Objectives:**

Hip abductor tendon tears remain an underrecognized diagnosis, initially classified under Greater Trochanteric Pain Syndrome. This often results in ineffective conservative treatment, providing only temporary pain relief. While certain surgical approaches, particularly knotless double‐row repair techniques (Hip Bridge) have shown promising clinical outcomes, comprehensive biomechanical data remain insufficient. Therefore, this study aimed to biomechanically compare Hip Bridge (HB) repair with the standard Mason‐Allen (MA) technique using a human cadaver model.

**Methods:**

Gluteus minimus and medius were released in 12 fresh‐frozen human cadaveric specimens and reattached to their anatomical footprints either with transosseous MA or knotless double‐row HB technique. HB consisted of two proximal PEEK (polyetheretherketone) anchors, each preloaded with double‐V shaped tapes, crossed, and distally fixated with two additional anchors. Femurs were fixated in a custom‐made sample holder while gluteal muscles were clamped using a cryo‐jaw. The construct underwent a cyclic loading test between 10 and 125 N for 150 cycles at 2.5 Hz (preload 10 N), followed by a pull‐to‐failure test. Failure mode and elongation were determined, the latter by a 3D optical measurement system. Statistical analysis was performed using *t*‐test.

**Results:**

HB repair resulted in significantly higher ultimate failure loads (339.1 ± 144.4 N) compared to the MA technique (209.6 ± 62.1 N, *p* = 0.0381). HB failed exclusively due to tendon failure, whereas MA exhibited different failure modes: tendon failure (1/6), bone cutting (4/6), and muscle rupture (1/6). During cyclic loading, the calculated final plastic elongation was 4.4 ± 0.5 mm for MA and 3.4 ± 1.4 mm for HB (*p* = 0.0731). During pull‐to‐failure testing, stiffness of 59.7 ± 12.5 N/mm (MA) and 66.8 ± 18.4 N/mm (HB) was observed (*p* = 0.247).

**Conclusion:**

The HipBridge technique provides superior biomechanical stability compared to the standard Mason‐Allen repair, showing significantly higher ultimate failure load and reduced failure variability. This advantage may be attributed to greater contact restoration of the anatomical footprint, which is particularly beneficial for treating weakened tendons and bones in elderly patients.

## Graphical abstract

Hip Bridge technique demonstrated significantly higher biomechanical failure load compared to the Mason‐Allen repair; with greater primary stability and reduced failure variability. This may be attributed to enhanced anatomical footprint restoration, which is particularly advantageous for weakened gluteal tendons in elderly patients.

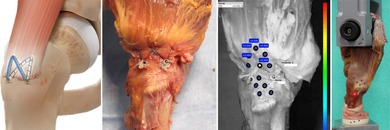

**Abbreviations**
GMax: M. gluteus maximus
GMed: M. gluteus medius
GMin: M. gluteus minimus
GTPS: great trochanteric pain syndrome
HB: HipBridge
MA: Mason‐Allen
PEEK: polyetheretherketon
THA: total hip arthroplasty
yrs: years

## 1 Introduction

In more than 50% of cases, greater trochanteric pain syndrome (GTPS) is caused by a manifest insufficiency of the gluteal muscles M. gluteus medius (GMed) and minimus (GMin) [1]. Its prevalence is indicated as approximately 10% in native hips, predominantly affecting patients over 60 years, with a remarkably higher incidence following (revision) total hip arthroplasty (THA) [2, 3]. Gluteal insufficiency is now recognized as a major contributor to persistent pain in THA patients [4]. However, hip abductor tendon tears remain underdiagnosed. Patients often have a long history of inefficient conservative treatment with only temporary pain relief [5]. In some cases, corticosteroid injections may further compromise tendon quality, adversely affecting surgical outcomes [6]. Delayed treatment can result in muscle atrophy and fatty degeneration, potentially leading to permanent disability and poor functional recovery [7].

Given the anatomical and functional similarities between the gluteal muscles and the shoulder rotator cuff, GMed, GMin, and parts of the gluteus maximus (GMax) have been described as the “rotator cuff of the hip” [2]. Consequently, repair techniques successfully implemented to the shoulder rotator cuff (SpeedBridge, Arthrex) were also adapted for the hip. The knotless double‐row Hip Bridge (HB) technique, described by the authors, offers a minimally invasive approach for broad‐based tendon reattachment to the anatomical footprint [3].

Preliminary studies have demonstrated mostly favorable short‐term results after double‐row repair of gluteal tendon tears [7, 8, 9, 10, 11]. In case of adequate patient selection and the surgeon's clinical experience, this technique when performed may lead to a stable reconstruction in 80%–90% of the interventions [9, 10, 11, 12]. In comparison to the shoulder, the hip abductor muscles endure significantly greater mechanical loads during daily activities [7]. Therefore, biomechanical stability is critical, particularly given the poor tissue quality in typically elderly patients. Despite its limitations, MA remains widely used due to its simplicity and cost‐effectiveness, though clinical outcomes vary [13]. This technique is therefore well‐suited for comparison. The lack of robust biomechanical data comparing different repair techniques—particularly MA versus newer methods—further complicates treatment decisions.

In 1994, Gerber et al. emphasized that mechanical stability is crucial for optimal tendon healing, requiring high initial fixation strength and minimal gap formation [14].

Therefore, the purpose of this cadaveric study was:

- to biomechanically compare the primary stability of the knotless double‐row technique (HB) with the established transosseous MA repair for the anatomical refixation of gluteal tendons [15],
- to assess construct performance based on cyclic elongation, stiffness, and ultimate load to failure, and
- to explore potential advantages of the HB technique regarding footprint restoration, reproducibility, and its applicability in elderly patients with reduced bone and tendon quality.

## 2 Materials and Methods

### 2.1 Ethical Considerations

Twelve fresh‐frozen human cadaveric hemi‐pelvic specimens were obtained from ScienceCare Inc. donor bank (Phoenix, Arizona, USA) to Arthrex GmbH (Munich, Germany). All tissue provided by ScienceCare is obtained with the appropriate informed consent of donors, in compliance with all applicable local, state, and federal laws and regulations governing the retrieval and supply of human tissue. ScienceCare maintains approved protocols, documented tissue sources, institutional review board (IRB) approval for tissue acquisition for research, and consent forms for tissue delivered. In accordance with the Local Ministry of Health regulations (Bayerische Landesärztekammer, Munich, Germany), this cadaveric study thus did not require additional local institutional review board approval. The authors declare that all experiments were performed according to the ethical principles of the Declaration of Helsinki.

### 2.2 Specimen Preparation/Hip Dissection

**FIGURE 1:**
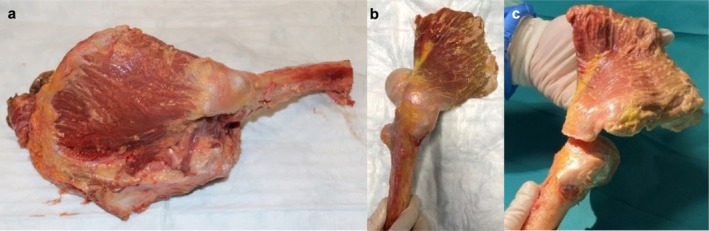
After coarse preparation of the cadaveric hemi‐pelvic specimens (a), dissections were performed only, preserving the gluteus medius and minimus tendon‐muscle unit (b). Afterwards, both parts were sharply released from their anatomic footprints (c).

Twelve fresh‐frozen human cadaveric hemi‐pelvic specimens were provided by ScienceCare Inc. donor bank. Selection criteria included 7 female and 5 male donors aged between 50 and 73 years (mean age 62 ± 7 years), a body mass index of 20.7–35.73 (mean BMI 27 ± 5) kg/m^2^ and no previous history of hip injury, surgery, chemotherapy, or radiation less than 1 year before death. Before coarse preparation, the specimens were matched between both repair groups based on their patient‐specific data. All specimens were stored at −20°C until the day before preparation and testing, where they were thawed at room temperature. After the coarse preparation with removing skin and soft tissue, the hemi‐pelvises were dissected, preserving only the GMed and GMin tendon‐muscle unit and joint capsule of the hip (Figure 1a,b). The tendons of both GMed and GMin were then sharply released from their anatomic insertion at the greater trochanter (Figure 1c). To minimize dehydration effects, all specimens were kept moist with saline solution during preparation and testing. Testing was conducted at room temperature and completed within 90 min per specimen. Cryo‐clamps were used for proximal muscle fixation, offering additional cooling at the muscle‐tendon interface.

### 2.3 Repair Technique

The specimens were then assigned to undergo either the established transosseous MA repair or the knotless double‐row HB repair for reattachment to their anatomical footprint. Both reconstruction techniques utilized the same number and type of anchors and sutures within their respective groups to ensure comparability.

### 2.4 Hip Bridge Technique

**FIGURE 2:**
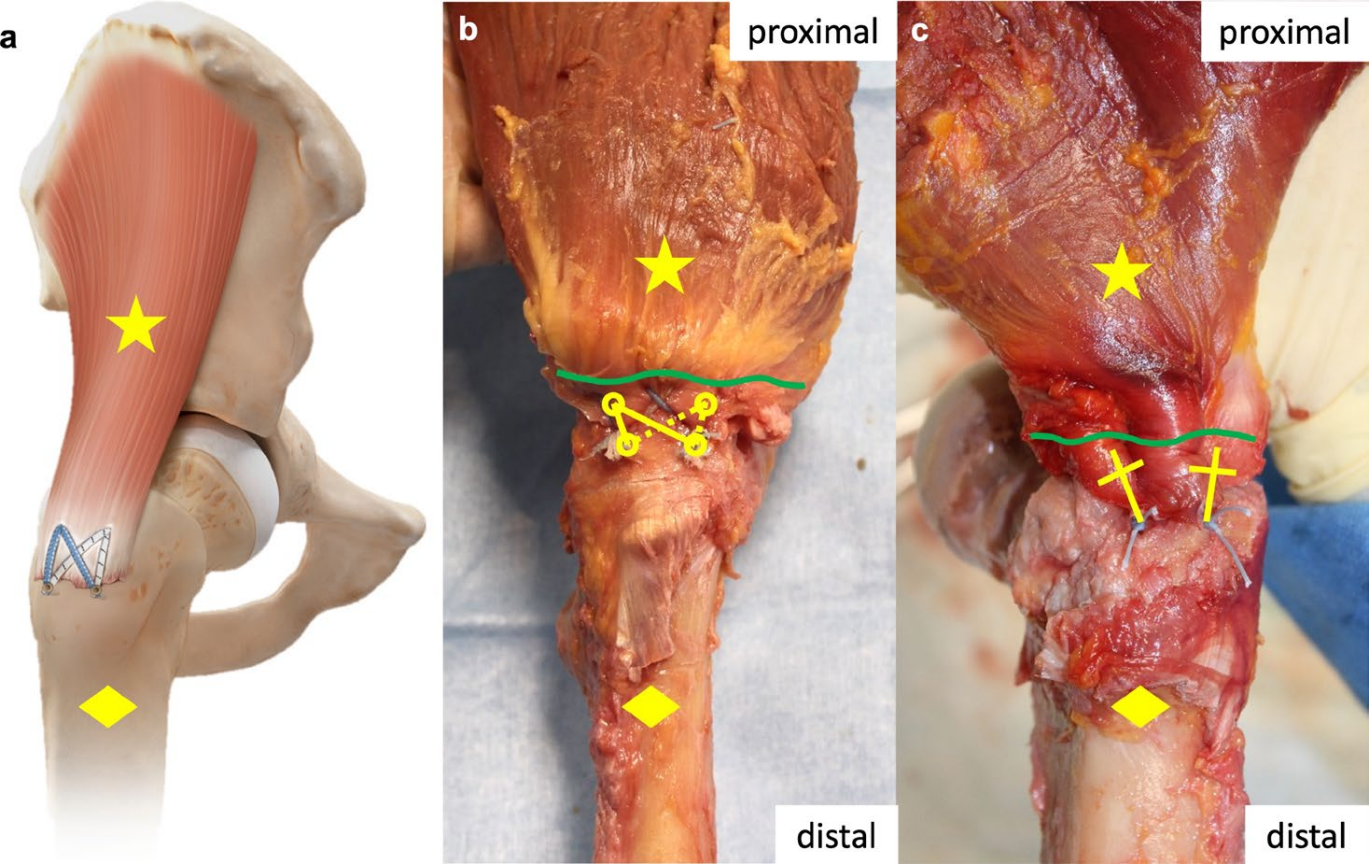
Repair techniques: Schematic illustration of HB technique (a). Knotless double‐row HB in double‐V shape (b). Modified MA suture technique (c). Circles mark anchor positions, lines suture pathes (FiberTape in HB, FiberWire in Mason‐Allen). Star = gluteal musculature, diamond = femur, green curved line = muscle tendon junction.

HB technique is a modification of the minimally invasive SpeedBridge technique (Arthrex), originally developed for rotator cuff repair in the shoulder [16]. It involves the placement of two proximal suture anchors (PEEK SwiveLock, 4.75 mm, Arthrex) at a 90° angle within the gluteal tendons' anatomical footprint. Each anchor is preloaded with a FiberTape (2 mm, Arthrex), which is subsequently shuttled through the gluteal tendons. For distal fixation, one FiberTape tail of each proximal anchor is retrieved in a crossed pattern and secured alongside the straight tails using two additional suture anchors (PEEK SwiveLock, 4.75 mm, Arthrex) (Figure 2a/b). While applying tension to the FiberTape sutures, the slack is reduced, and the tendons are compressed against the bone. The distal anchors are then inserted into newly prepared bone sockets approximately 20 mm distal to the first row. In all cases, the anatomical footprint was completely restored to ensure optimal biomechanical stability.

### 2.5 Mason‐Allen Technique

In this study, the transosseous standard MA technique as the most commonly used single‐row configuration was performed [14]. A free needle looped size S loaded with FiberWire (FiberWire, 2 mm, braided polyblend, Arthrex) was utilized. After piercing through the cortical bone (width approx. 6–8 mm, depth approx. 3–5 mm) both suture tails were shuttled through the gluteal tendons and knotted in a MA‐typical crossed pattern to minimize the risk of tendon cut‐through. This was followed by 5 half surgeons knots (Figure 2c). MA‐repair was performed twice with a distance of 20 mm.

### 2.6 Biomechanical Testing

**FIGURE 3:**
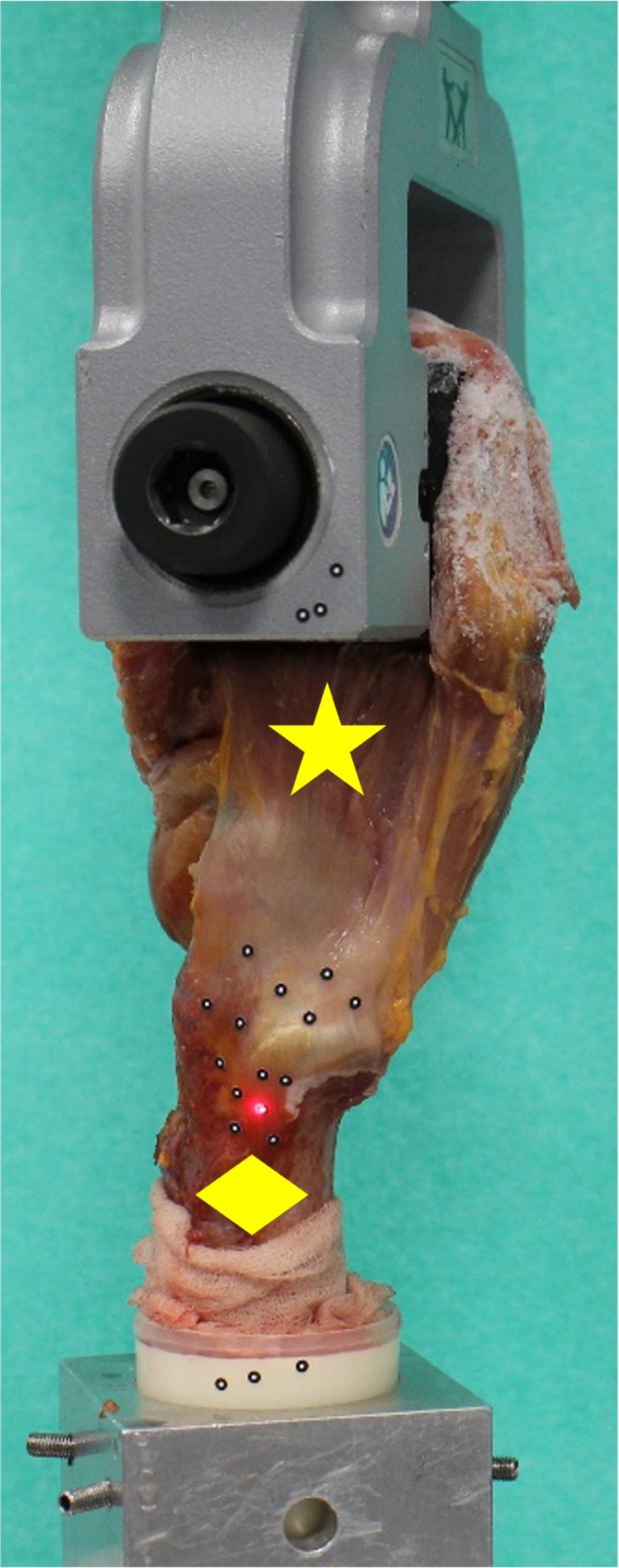
Femoral side (diamond) of the cadaveric hemi‐pelvic mounted in a material testing machine, while the gluteal muscles (star) are fixed using cryo‐clamps and optical circular markers are set.

Following specimen preparation, the femur was potted using a two‐component polyurethane (RenCast FC 52/53 Isocyanate/FC 53 Polyol, Huntsman Advanced Materials GmbH, Switzerland) and placed in a custom‐made sample holder. After mounting on the baseplate of a materials testing machine (ElectroPuls E10000, Instron) the gluteal muscles were clamped using a cryo‐jaw (Figure 3). Afterwards, two sets of circular markers containing at least five measurement points were glued to the gluteal muscles and greater trochanter, respectively.

The test protocol was adapted with modifications according to Dishkin‐Paset et al. [17], who utilized a comparable cyclic loading setup to assess gluteal tendon repairs. Similar parameters were also used in rotator cuff studies according to Pauly et al. and Nelson et al. [17, 18], justifying the selected load range, frequency, and number of cycles to simulate early postoperative mechanical demands. The loading direction was aligned vertically from distal to proximal along the gluteal muscle axis, simulating the tensile force vector of the abductors during contraction. Although this does not fully replicate dynamic in vivo loading conditions, it ensures standardized and reproducible comparison of primary repair strength.

**FIGURE 4:**
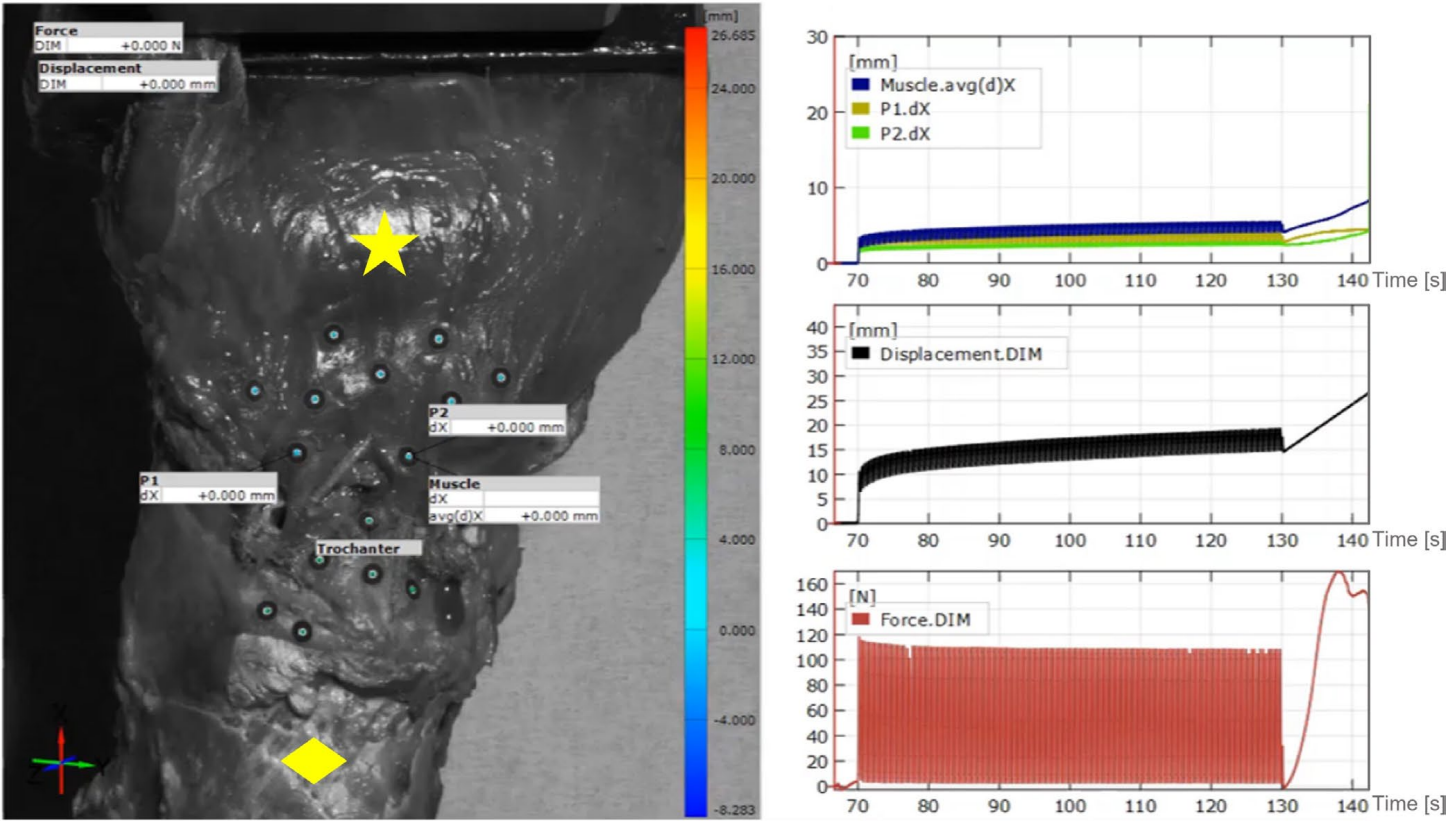
Elongation (mm) and displacement (mm) weremeasuredat different times (s) using a GOM ARAMIS 3D optical tracking system (150 Hz; Carl Zeiss GOM Metrology GmbH, Braunschweig, Germany), enablingprecisethree‐dimensional analysisbetweenmuscle (star) and greatertrochanter (diamond) via reflective markers.

A preload of 10 N was then applied before the construct was subjected to the test protocol with modifications according to Dishkin‐Paset et al. [17]. The test protocol contained cyclic loading between 10 and 125 N for 150 cycles at 2.5 Hz, followed by a pull‐to‐failure test at a speed of 1 mm/s. Meanwhile, load and displacement were recorded by the standard measuring software of the materials testing machine (WaveMatrix, Instron). Elongation was measured using a GOM ARAMIS 3D optical tracking system (150 Hz) (Carl Zeiss GOM Metrology GmbH, Germany), which enabled precise three‐dimensional displacement analysis between muscle and greater trochanter using reflective marker points (Figure 4).

Primary outcome measures included:

- Plastic elongation (mm): irreversible deformation between the muscle and greater trochanter at the final loading cycle, representing gap formation.
- Plastic/elastic elongation (mm): maximum reversible and irreversible displacement at the last cycle.
- Construct stiffness (N/mm): measured during the linear region of the load–displacement curve during pull‐to‐failure.
- Ultimate failure load (N): maximum load sustained before construct failure.

Secondary outcome:

- Clinical failure threshold was defined as ≥ 5 mm of plastic elongation, based on prior work [16].

### 2.7 Statistical Analysis

Data analysis was performed using MATLAB (MATLAB R2023a, MathWorks, USA). Statistical evaluations were conducted with SigmaPlot (SigmaPlot Version 15, Systat Software GmbH, Germany). Normal distribution of all continuous variables was confirmed using the Shapiro–Wilk test. Subsequently, parametric one‐tailed *t*‐tests were used to compare outcome parameters between the Hip Bridge and Mason‐Allen groups (significance set at *p* ≤ 0.05). Categorical and continuous variables are shown as count and mean ± standard deviation.

## 3 Results

### 3.1 Failure Mode

One specimen treated with the HB technique experienced immediate failure due to slippage of the reattached gluteal muscles and translation of the posterior bone anchor, likely attributable to insufficient tissue quality and was therefore excluded from further evaluation. This led to a final group size of *n* = 5 (HB) and *n* = 6 (MA) repair technique. While all HB reconstructions failed exclusively at the tendon, MA constructs demonstrated three different failure modes: tendon failure (1/6), bone cutting (4/6), and muscle rupture (1/6).

### 3.2 Plastic/Elastic Elongation

**FIGURE 5:**
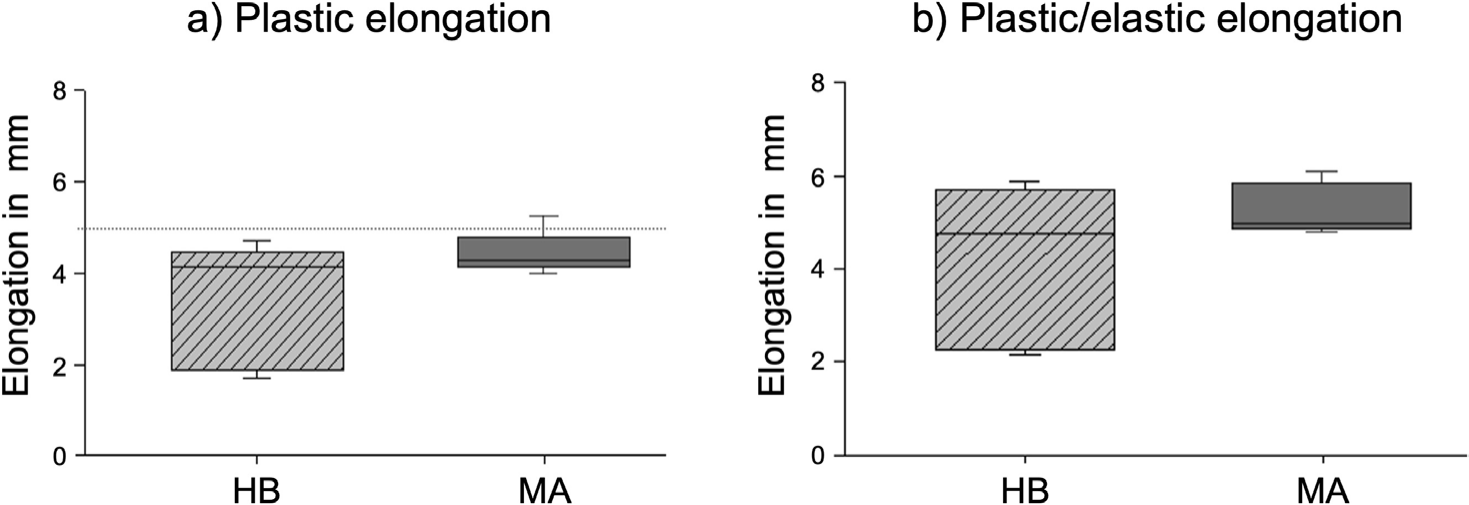
Boxplots demonstrate final plastic (a) and plastic/elastic (b) elongation of HB and MA in mm.

Using a 3D camera system (GOM ARAMIS) specific elongation of the repair construct was analyzed, with particular focus on the critical muscle/tendon to bone interface (Figure 4). The final plastic elongation measured 4.4 ± 0.5 mm in the MA and 3.4 ± 1.4 mm in the HB group (*p* = 0.0731). Notably, none of the HB constructs exceeded the critical 5 mm threshold, whereas one MA construct did, which was classified as clinical failure [18]. The plastic/elastic elongation, representing the maximum construct elongation after the last cycle, reached 5.3 ± 0.5 mm for the MA and 4.1 ± 1.7 mm for the HB group (Figure 5).

### 3.3 Maximum Failure Load

**FIGURE 6:**
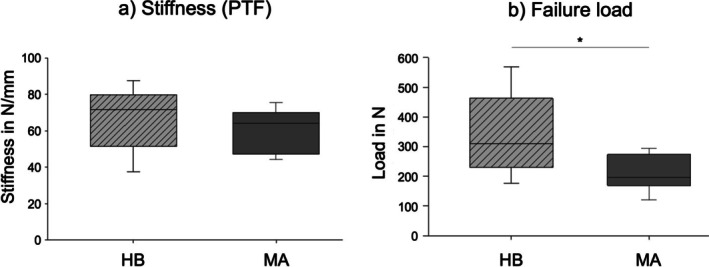
Boxplots demonstrate stiffness (N/mm) at pull to failure time (a) and failure load in N (b) for HB and MA.

During pull‐to‐failure testing, refixation stiffness was measured at 59.7 ± 12.5 N/mm for MA and 66.8 ± 18.4 N/mm for HB technique (*p* = 0.247). Most importantly, the ultimate failure (major parameter of primary stability) was significantly higher in the HB group compared to the MA group (339.1 ± 144.4 vs. 209.6 ± 62.1 N, respectively; *p* = 0.0381) (Figure 6). All results are summarized in Table 1.

**TABLE 1:** Results of biomechanical testing.

	Final elongation		Pull‐to‐failure	
	Plastic in mm	Plastic/elastic in mm	Stiffness in N/mm	Ultimate load in N
HipBridge	3.4 ± 1.4	4.1 ± 1.7	66.8 ± 18.4	339.1 ± 144.4*
Mason‐Allen	4.4 ± 0.5	5.3 ± 0.5	59.7 ± 12.5	209.6 ± 62.1*
*p*‐value	0.0731	0.104	0.247	**0.0381**

*Note:* The ultimate load is significant at 0,0381 (in bold).

* *p* ≤ 0.05 (*t*‐test with one‐tailed *p*‐value).

## 4 Discussion

This biomechanical cadaveric study demonstrated that the knotless double‐row HipBridge repair technique showed a significantly higher ultimate failure load compared to the traditional Mason‐Allen transosseous repair. Additionally, the HB construct exhibited trends toward greater construct stiffness and reduced elongation under cyclic loading, suggesting improved primary stability under simulated postoperative loading conditions.

The aim of this study was to compare the innovative HB technique with the widely used MA repair, the most common and convenient technique for gluteal tendon refixation.

### 4.1 Comparison of Biomechanical Stability Between HB and MA Techniques

Our findings demonstrate that the HB technique exhibited a significantly higher ultimate failure load compared to the conventional MA repair. Additionally, HB showed less elongation during cyclic loading and a higher stiffness during the pull‐to‐failure test for the HB group, although this did not reach statistical significance. The significantly higher ultimate failure load and reduced elongation observed with HB imply a lower risk of early postoperative gapping and construct failure. Clinically, this may translate into a safer initiation of early active‐assisted and weight‐bearing protocols, potentially shortening rehabilitation timelines and improving patient confidence during recovery.

### 4.2 Construct Performance and Clinical Relevance

Plastic elongation, defined in this study as irreversible deformation during cyclic loading, has been linked in literature to stress softening, characterized by a progressive decrease in tensile modulus prior to failure [19]. This inelastic behavior compromises the tendon's ability to absorb energy and return to its unloaded state, potentially leading to early failure, rupture, muscle insufficiency, or tendinopathy [20]. Given its irreversible nature, plastic elongation in this study was equated to gap formation [19]. This highlights the importance of strong primary stability, particularly in elderly patients with poor tendon quality, to prevent prolonged immobilization and promote solid healing [14]. Given that gluteal tendon tears predominantly affect older individuals with compromised tendon and bone quality, the broad‐based footprint compression achieved by the double‐row HB construct addresses these challenges directly. We anticipate that HB may offer superior outcomes in patients with poor bone stock or chronic tendon degeneration, where single‐row repairs often fail. These findings are crucial for early functional rehabilitation, as tendon detachments frequently occur in the early postoperative period, before bone‐to‐tendon healing is complete [20].

The MA technique remains a cost‐effective option that requires no special equipment, making it widely used for multiple tendon refixations [21]. Gerber et al. found the modified MA suture to be superior among conventional techniques, citing its broad‐based fixation and minimal strangulation [14]. Although our study used an open approach, the HB technique is easily translatable to minimally invasive endoscopic procedures, allowing the benefits of improved biomechanics without increased surgical morbidity. While the use of four anchors may raise initial implant costs, this could be offset by lower complication and revision rates, yielding overall cost savings in the long term.

### 4.3 Failure Modes, Biological Healing, and Future Directions

A key finding of our study was that HB constructs failed exclusively at the tendon, whereas MA repairs demonstrated three distinct failure modes: tendon failure (1/6), bone cutting (4/6) and muscle tear (1/6). This further supports the superior primary stability of the reattached footprint after HB repair, as it restores the anatomical footprint more effectively [22]. As an explanation, the advantage of broad‐based anatomical footprint reconstruction using HB over the punctual rigid fixation (MA) enables not only a stable and reproducible refixation of the gluteal tendon from the beginning, but possibly even more important, a good mid‐ to long‐term biological healing. The homogeneous failure mode (tendon failure only) and lower variability with HB suggest more predictable biomechanical performance. In a clinical setting, this consistency could decrease the incidence of mechanical complications—such as anchor pullout or suture cut‐through—and thus reduce retear rates and the need for revision surgery, especially in osteopenic bone typical of elderly patients. Barrera et al. determined that mini‐open repair of chronic gluteus medius tendon tears using a double‐row technique showed good clinical and functional outcomes at short follow‐up (secondary stability) [23]. Depending on our clinical experience, the HB technique shows good results also regarding secondary stability.

Biomechanical data on the different repair techniques for rotator cuff repair of the shoulder are well investigated, but data for gluteal tendon repair are still rare. Some studies could demonstrate that double‐row repair had a higher ultimate failure load when comparing with single‐row techniques for rotator cuff repair of the shoulder [24]. To the best of our knowledge, Kahlenberg et al. conducted the only biomechanical cadaveric study comparing single‐ versus double‐row technique for gluteal tendon repair after hip abductor tears. However, MA repair was not included and compared. They demonstrated that double‐row suture repair yields improved footprint coverage and a mean but not significant higher yield load [7]. Comparing both studies, we used a state‐of‐the‐art technique for measuring the elongation, 3D optical measurement technique (150 Hz) vs. digital video (60 Hz). In addition, our mean values regarding failure load and stiffness were higher in the HB group. This could be interpreted as an indication of superior stability. However, we could only detect trends regarding less elongation after cyclic loading when using the HB technique. A larger sample size may be required to detect significant differences in plastic elongation.

While the applied uniaxial loading protocol reflects standard practice in biomechanical tendon studies, it does not fully replicate the complex multi‐vector forces acting on the hip abductors in vivo. Future studies incorporating multi‐axial loading patterns and dynamic simulations may provide even more clinically relevant insights.

Future clinical trials should compare patient‐reported outcomes, retear rates (via postoperative imaging), and rehabilitation metrics between HipBridge and Mason‐Allen repairs. Additionally, exploring the role of HB in partial‐thickness or chronic degenerative tears may further establish its utility in diverse clinical scenarios.

### 4.4 Strengths and Limitations

#### 4.4.1 Strengths

This study offers several notable strengths. First, a high‐resolution 3D optical tracking system (150 Hz) was used to precisely capture elongation and construct displacement, providing superior measurement accuracy compared to conventional video‐based analysis. Second, the standardized and reproducible loading protocol was adapted from validated models in both gluteal and rotator cuff tendon repair research, ensuring methodological comparability. Third, we used fresh‐frozen human cadaveric specimens with consistent preparation and fixation techniques to closely simulate physiological conditions. Lastly, this is, to our knowledge, the first biomechanical cadaveric study directly comparing knotless double‐row HipBridge repair with the established Mason‐Allen technique, offering valuable insights for both open and minimally invasive surgical applications.

#### 4.4.2 Limitations

The comparison of both surgical techniques was conducted in a biomechanical test setup, allowing for the assessment of primary stability only, immediately following the procedure. Additionally, statistical significance was not reached for elongation and stiffness. An a priori power analysis based on the primary outcome parameter (ultimate failure load) justified a minimum of five specimens per group. A post hoc analysis confirmed that the observed difference yielded a statistical power of 0.81 (Cohen's *d* = 1.14), supporting the robustness of our primary finding. While the sample size was limited due to the high cost and restricted availability of human cadaveric specimens, it remains consistent with previous biomechanical studies in this field [7]. Despite this limitation, the significant difference in ultimate failure load indicates that the primary conclusion is reliable. However, future studies with larger sample sizes are warranted to validate and generalize these results and to further evaluate the effects of different repair techniques on secondary biomechanical parameters such as elongation and stiffness. Furthermore, our data showed a greater variance in the HB group. This might be due to a reduced tissue quality caused by the freezing process or the slight imbalance regarding gender. Possible differences in tendon quality between specimens have also been reported in previous biomechanical studies, such as that by Kahlenberg et al. [7]. Due to the test setup and the biomechanical setting of the study, both repair techniques were performed as open and not endoscopic procedures. However, Maslaris et al. demonstrated that in clinical practice open and endoscopic gluteal tendon refixation yield comparable outcomes [25]. Nevertheless, our results cannot be completely generalized to endoscopic abductor tendon repair although the newly analyzed HB technique is feasible as both an endoscopic and open procedure. Additionally, a bone mineral density assessment was not conducted. The intact tendon was not biomechanically tested, as double testing was not possible due to tissue degradation and the need for identical initial conditions.

## 5 Conclusion

Compared to the established and widely used Mason‐Allen technique, knotless double‐row fixation with four anchors (HipBridge technique) demonstrated significantly higher biomechanical failure load. Additionally, HB repair restores the broad‐based anatomical footprint, providing a stable and reproducible refixation of the gluteal tendon mirrored by reduced failure variability, which may enhance biological healing. Our clinical experience supports the biomechanical superiority of the HB technique; however, further studies are required to validate the significance of these findings.

## Author Contributions

**Vanessa Twardy:** data curation, formal analysis, resources, software, visualization, writing – original draft, writing – review and editing. **Daniela Warnecke:** data curation, formal analysis, investigation, methodology, resources, software, visualization, writing – original draft. **Peter M. Prodinger:** conceptualization, funding acquisition. **Norbert Harrasser:** conceptualization, investigation. **Christian Scheele:** methodology. **Rüdiger von Eisenhart‐Rothe:** supervision. **Martina Roth:** data curation, investigation, methodology, software. **Ingo J. Banke:** conceptualization, formal analysis, funding acquisition, investigation, methodology, project administration, resources, validation, visualization, writing – review and editing, writing – original draft.

## Ethics Statement

An ethical waiver (“Anatomical Materials Transfer Agreement”) is concluded between ScienceCare Inc. and Arthrex GmbH. All tissue provided by ScienceCare is obtained with the appropriate informed consent of donors, in compliance with all applicable local, state, and federal laws and regulations governing the retrieval and supply of human tissue. ScienceCare maintains approved protocols, proof of tissue sources, institutional review board approval for tissue acquisition for research, and consent forms for tissue delivered. In accordance with the Local Ministry of Health regulations (Bayerische Landesärztekammer, Munich, Germany), this cadaveric study does not require additional local institutional review board approval. The authors declare that all experiments were performed according to the ethical principles of the Declaration of Helsinki.

## Consent

The authors have nothing to report.

## Conflicts of Interest

I.J.B. is a consultant for Arthrex; D.W. and M.R. are Senior Research Engineers of Arthrex. The other authors declare that they have no competing interests.

## Data Availability

Datasets used and/or analyzed during the current study are available from the corresponding author on reasonable request.
